# Complete Genome Sequence of Escherichia coli AS19, an Antibiotic-Sensitive Variant of E. coli Strain B REL606

**DOI:** 10.1128/genomeA.00385-18

**Published:** 2018-05-03

**Authors:** Mariana Avalos, Marten Boetzer, Walter Pirovano, Nelson E. Arenas, Stephen Douthwaite, Gilles P. van Wezel

**Affiliations:** a Institute of Biology, Leiden University, Leiden, The Netherlands; b Bioinformatics Department, BaseClear BV, Leiden, The Netherlands; c Department of Biochemistry and Molecular Biology, University of Southern Denmark, Odense, Denmark

## Abstract

The chemically mutagenized Escherichia coli strain AS19 was isolated on the basis of its enhanced sensitivity to different antibiotics, in particular to actinomycin. The strain was later modified to study rRNA modifications that confer antibiotic resistance. Here, we present the genome sequence of the variant E. coli AS19-RrmA^-^.

## GENOME ANNOUNCEMENT

Escherichia coli is a bacterium commonly found in the human intestine as well as in other animals and, like other bacteria, pathogenic strains can rapidly acquire antibiotic resistance and become life-threatening. The emergence of antibiotic resistance is a worldwide problem that reduces or inhibits the efficacy of antibiotics, putting the lives of millions of people at risk (1). With failing drug discovery efforts, new approaches are required to find drug targets (2). Obtaining insights into the genetics of antibiotic resistance is therefore of utmost importance.

E. coli strain AS19 is an actinomycin-sensitive strain that was selected to study antibiotic sensitivity and how this might be linked to cell permeability. The strain was obtained by chemical mutagenesis of E. coli strain B with *N*-methyl-*N*′-nitroso-*N*-nitroguanidine (3). AS19 has been used further in studies of bacteriophage infection (4) and antibiotic resistance/sensitivity (5). In a more recent study, the derivative E. coli AS19-RrmA^-^ was developed to examine how rRNA modifications affect susceptibility to macrolide-lincosamide-streptogramin B (MLS_B_) and ketolide antibiotics (6). Strain AS19-RrmA^-^ harbors a kanamycin resistance cassette disrupting the rRNA methyltransferase gene *rrmA* (recently renamed to *rlmA*) and retains its sensitivity to actinomycin and to erythromycin, tylosin, and novobiocin. We have sequenced the E. coli strain AS19-RrmA^-^ in order to identify genes related to antibiotic resistance/sensitivity.

Genomic DNA was isolated from E. coli strain AS19-RrmA^-^, as described elsewhere (7), and was sequenced using the Illumina HiSeq and PacBio RS machines by BaseClear BV (Leiden, The Netherlands). The quality of the Illumina FASTQ sequences was enhanced by trimming low-quality bases using the “Trim sequences” option of CLC Genomics Workbench version 7.0.4 (Qiagen Bioinformatics). The quality-filtered sequence reads were assembled into a number of draft contig sequences using the “*de novo* assembly” option of CLC Genomics Workbench version 7.0.4. The optimal *k-*mer size was automatically determined using KmerGenie (8). The draft contigs were aligned to the PacBio continuous long reads (CLR) using BLASR (8) and subsequently scaffolded into a single chromosome using the SSPACE-LongRead scaffolder version 1.0 (9). The remaining gaps were closed in an automated manner using GapFiller version 1.10 (10).

E. coli AS19-RrmA^-^ has a chromosome of 4,607,960 bp, with a GC content of 50.78%. The genome contains 4,300 genes, 3,597 of which have a known function, and it specifies 76 tRNAs and 21 rRNAs. The genome was compared to the genome of E. coli B strain REL606. In total, 660 mutations were found, from which 180 mutations resulted in amino acid changes. Mutations related to drug efflux pumps were found among the relevant mutations. We are currently analyzing these mutations to uncover the genetic cause of the enhanced antimicrobial sensitivity.

### Accession number(s).

The complete sequence of the genome of E. coli AS19-RrmA^-^ has been deposited in GenBank under the accession no. CP027430.
